# A cross-sectional analysis of county-level determinants of pandemic-era policy implementation

**DOI:** 10.3389/fpubh.2026.1797664

**Published:** 2026-06-19

**Authors:** Natasha J. Williams, Melanie F. Molina, Amy Yunyu Chiang, Soo Park, Matthew Brandner, Madelaine Faulkner Modrow, Paul Meissner, John Kornak, Heather Kitzman, Carmen R. Isasi, Alan F. Kaul, Djeneba Audrey Djibo, Sylvia Sudat, Jaime Orozco, Janna Garcia Torres, Vinit P Nair, Pelin Ozluk, Thomas Carton, Mark J. Pletcher, Rita Hamad

**Affiliations:** 1 NYU Langone Health, Department of Population Health, Institute for Excellence in Health Equity, New York, NY, United States; 2 Department of Emergency Medicine, University of California, San Francisco, San Francisco, CA, United States; 3 Department of Medicine, Division of Clinical Informatics and Digital Transformation, University of California, San Francisco, San Francisco, CA, United States; 4 Action Research Center for Health, University of California, San Francisco, San Francisco, CA, United States; 5 UCSF Division of General Internal Medicine, Department of Medicine at the San Francisco General Hospital and Trauma Center, University of California, San Francisco, San Francisco, CA, USA; 6 Department of Epidemiology and Biostatistics, University of California, San Francisco, San Francisco, CA, United States; 7 Albert Einstein College of Medicine / Montefiore Medical Center, Bronx, NY, United States; 8 Parkland Health, Dallas, TX, United States; 9 Department of Epidemiology and Population Health, Albert Einstein College of Medicine, Bronx, NY, United States; 10 Medical Outcomes Management, Inc., Sharon, MA, United States; 11 Epidemiology & Science, CVS Healthspire Payor & Life Science Solutions, Blue Bell, PA, United States; 12 Sutter Health, San Francisco, San Francisco, CA, United States; 13 Department of Population Health, NYU Grossman School of Medicine, New York, NY, United States; 14 G19 Studio, Chicago, IL, Sharon, MA, United States; 15 Elevance Health Inc., Indianapolis, IN, United States; 16 Louisiana Public Health Institute, New Orleans, LA, United States; 17 Department of Social and Behavioral Sciences, Harvard School of Public Health, Boston, MA, United States

**Keywords:** health policy, policy evaluation, public health, COVID-19, pandemic

## Abstract

**Introduction:**

Local COVID-19 policies are increasingly recognized as determinants of variation in disease transmission and other health outcomes. There is significant geographic variability in policy implementation, but little understanding of factors contributing to policy variation.

**Data and methods:**

Using data from the US COVID-19 County Policy Database, we analyzed correlates of county-level policy implementation (*N* = 309 counties in 50 states across 2020–2021). Using multivariable regression, we examined the association of county characteristics with policy comprehensiveness overall and across three policy domains (containment/closure, economic support, public health). We focused on county characteristics capturing sociodemographic, political, and environmental factors that may represent predictors of policy adoption.

**Results:**

Urbanicity was positively associated with increased adoption of more public health policies, Democratic voter percentages were positively associated with increased adoption of all policies, and average temperature was negatively associated with adoption of all policies.

**Conclusion:**

These findings provide insight into the possible predictors of the variation in local policy implementation during the COVID-19 pandemic, as well as informing future policy research and public health crisis management.

## 1 Introduction

State and local authorities have constitutional authority to proactively enact measures to protect public health and control the spread of disease within their jurisdictions. This decentralized policymaking approach allows responses to be tailored to local context, but it also creates substantial variation in policy environments across jurisdictions and may complicate efforts to address public health crises equitably and effectively. As a result, geographic variation in policies also may contribute to geographic disparities in health and related social conditions (1–8).

Throughout the COVID-19 pandemic, states exercised their authority to develop policies designed to curtail viral spread and protect the population. These pandemic-era policies included containment and closure policies (e.g., limiting the number of people at events) as well as public health measures (e.g., mandatory masking and contact tracing) (9). Counties and other local jurisdictions also implemented policies, including economic response policies intended to reduce the hardship associated with the pandemic and related mitigating measures (9). Together, these policies shaped local public health measures and containment efforts. As a result, states may have been able to effectively manage the impact of the pandemic on their communities and minimize the impact on the health care systems (10–13).

**Figure 1:**
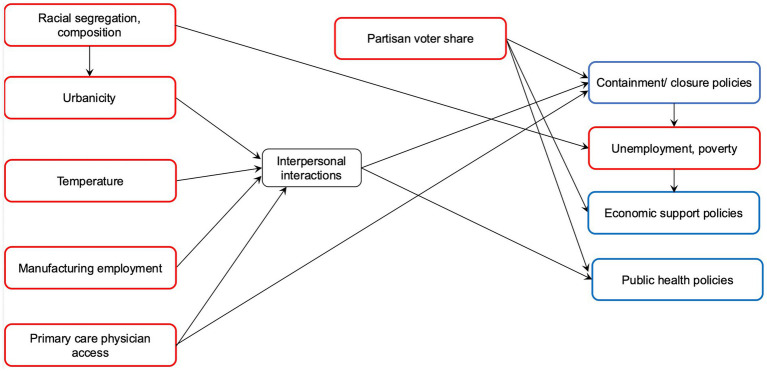
Conceptual diagram linking county characteristics with county policies. Blue boxes indicate primary dependent variables, and red boxes indicate independent variables in the models presented below. Hypothesized variables that contribute to county-level comprehensiveness policies.

Prior studies have demonstrated that political polarization among states and government leaders is strongly associated with implementing social policy and with public responses to those policies (14, 15). Studies have shown that state-level factors including more partisan political control are related to passage of health legislation like obesity-related bills (16, 17), abortion restrictions, health insurance coverage, and health outcomes (15, 18, 19). Similar patterns persisted during the COVID-19 pandemic, with partisanship predicting school re-opening plans (20). Given this, there are compelling reasons to expect that pandemic-era policy implementation could be related to multiple local factors. More broadly, there are strong reasons to expect that pandemic-related policy comprehensiveness would vary according to multiple county characteristics, including political composition, healthcare resources, economic structure, and geography (see Figure 1 for the conceptual diagram linking county characteristics with county policies). For example, social determinants of health and concentration of vulnerable populations may have shaped local policy comprehensiveness. Early in the pandemic, the 20% of U. S. counties with majority Black populations accounted for 52% of COVID-19 diagnoses and 58% of COVID-19 deaths (21). Further, even before the pandemic, segments of the population had fewer public health and economic resources on average (22), such that policymaking may have been less robust in these areas.

Earlier work characterized county-level pandemic-era policies and documented substantial variation both within and across states (9, 23). However, the factors contributing to this variation in policy implementation have not been systematically quantified at a national level. Few studies have examined key drivers of policy comprehensiveness across multiple policy domains at the county level. Understanding the variability in policy implementation between counties has implications not only for future pandemic preparedness, but also for understanding how local policy environments may contribute to broader public health challenges.

To help fill this gap in the literature, our study used a novel policy database to examine correlates of county-level pandemic-era policies including containment and closure policies, economic response policies, and public health policies. Identifying county characteristics associated with more or less comprehensive policy response may inform public health crisis management and preparedness and improve understanding of how local contexts shape policymaking during public health emergencies.

## 2 Materials and methods

### 2.1 Data

Data were drawn from the U. S. COVID-19 County Policy (UCCP) Database, a novel data set that includes weekly data on 26 policies from January 2020 to December 2021. The UCCP Database includes 309 counties across 50 states and Washington, D.C. (Supplementary Table A). While there are 3,143 counties (or county equivalents) in the U. S. and Washington, D.C., these jurisdictions represent over half of the U. S. population and are diverse with respect to geography, demographics, and partisanship.

The development of the database has been described in depth in prior work (9). Briefly, counties were selected for inclusion in the UCCP Database randomly using probability-proportional-to-size sampling, with overrepresentation of counties ranked highly on the CDC’s Social Vulnerability Index. The county policy data collection tool was based on a similar international tool used by the Oxford COVID-19 Government Response Tracker (24), and UCCP data collectors gathered county policy data from a variety of sources, including government websites, policy and government response summaries and databases, press releases, news articles, and social media posts by government organizations.

#### 2.1.1 Outcomes: county policies

The UCCP Database includes data on 26 policies in three policy domains. These include (1) containment and closures policies, (e.g., workplace closures, canceling of public events); (2) economic response policies, (e.g., housing and income supports); and (3) public health measures, (e.g., face covering mandates, vaccination availability).

For each policy, data collectors abstracted the comprehensiveness of the policy in a given week and its source documentation. Data were collected and stored using the secure Research Electronic Data Capture (REDCap) data entry and management platform (25). Despite a thorough search of multiple sources, in some cases there was no clear documentation on the county policies of interest for a given week. For these, missing county data for a given week was filled in with the corresponding state policy for that week. If there was no information on the policy at either the county or the state level, we coded the policy as missing and assume that there was no policy in place for those counties. While this assumption may not always be accurate, if policies were difficult for our trained staff to find after a systematic search, they were also likely to be difficult for residents to locate, resulting in no substantive restrictions in place. For example, counties with no clear documentation of a face covering policy were coded the same as those with clear documentation of no face covering requirement. We also did not capture more nuanced policy scenarios, for example, in which a county policy is in contradiction with (or weaker than) a state policy; such scenarios are outside the scope of the current analysis but can be examined in future research. Further details on data collection are provided in the Supplementary material.

We then created an index of policy comprehensiveness for each county in each week. To allow for greater variation and nuance in county policy landscapes, rather than simply tallying the number of policies implemented, we calculated an overall index of comprehensiveness ranging from 0 to 1, with 0 representing no policy in place, 1 representing maximal comprehensiveness, and intermediate categories represented by fractions thereof. For example, for public events, no restriction was coded as 0, minimal (≥ 50% capacity) limitations was 0.25, major (< 50% capacity) limitations was 0.50, recommended cancellation was 0.75, and required cancellation was 1. For the three policy domains (containment and closure, economic support, and public health), we derived a domain-specific comprehensiveness score by summing the scores for policies within that domain, which reflect the cumulative breadth of local policy response (23). We then summed the comprehensiveness scores from the three policy domains to compute an overall policy comprehensiveness score. These scores were created for each week throughout the study period for each county. The potential range of overall policy comprehensiveness scores was 0 to 27, with non-integer values possible.

Finally, to create our primary outcomes, we calculated the *mean* domain-specific and overall policy comprehensiveness scores for each county over the entire study period (January 2020 to December 2021). As a sensitivity analysis, we also derived the *maximum* domain-specific and overall policy comprehensiveness scores for each county over the same period. Our focus in this analysis is on these summary measures of overall policy comprehensiveness, rather than on the detailed temporal dynamics of policy trajectories over time.

#### 2.1.2 Predictors: county characteristics

For the predictors, we selected pre-pandemic county characteristics that could plausibly be linked to county policy implementation based on prior theoretical or empirical work, as described in the Introduction. We extracted county-level characteristics from publicly available data sources from the year 2019 or as close to 2019 as possible to ensure that they preceded the COVID-19 pandemic onset and would therefore be predictive of COVID-19-related policies, rather than consequential to them. We then merged them with the UCCP Database using county geocodes. These included poverty rate (percent of people of all ages in poverty, 2019), (U. Bureau) percentage White (2015–2019), (U. Bureau) percent urban, (U. Bureau) racial dissimilarity index, which measures the percentage of one group that would have to move across neighborhoods to be distributed the same way as the second group (26), (2015–2019), Democratic voter percentage,(U. Bureau) average annual temperature (2019), primary care physicians per 100,000 residents (2019), was rescaled to 10 million residents to facilitate comparison across predictors in regression models, while retaining the same underlying county level-measure and number of jobs in manufacturing (2019). We then merged these county-level characteristics into the UCCP Database using county Federal Information Processing System (FIPS) codes. We used regression imputation to fill in any missing county characteristics. For each county-level predictor, the number and proportion of missing observations prior to imputation were reported; only two county-level variables had missing data and that each had fewer than 1% of counties missing (Supplementary Table B). Given missing data on county characteristics were minimal, we expect this to have a negligible impact on our findings, as fewer than 1% of counties were missing data for the imputed variables. For variables derived from the American Community Survey (ACS), we used 5-year estimates from 2015–2019. One-year ACS estimates are only available for areas with populations 65,000 or more, and they are not recommended for smaller geographies (27). Since we are using granular county areas and some U. S. counties have fewer than 65,000 people, we follow the Census recommendation to use ACS 5-year estimates.

### 2.2 Statistical analysis

We generated descriptive statistics of the predictors and comprehensiveness scores. To check the assumption of independence and assess collinearity among covariates, we calculated Pearson’s correlation coefficients between each pair of predictors (Supplementary Table C). We then used multivariable linear models, regressing overall and domain-specific policy comprehensiveness scores on the county characteristics described above. We selected covariates based on theoretical and empirical literature (e.g., partisanship, healthcare/resources). Given the UCCP sample was selected using a probability-proportional-to-size sampling strategy and there are no publicly available survey weights that would support nationally representative inference, we did not apply sampling weights in our regression models. As a sensitivity analysis, we also examined the association between the same county characteristics and the *maximum*, as opposed to mean, domain-specific and overall policy comprehensiveness scores across the study period. All analyses were conducted using R, and two-sided *p*-values <0.05 were considered statistically significant.

### 2.3 Ethical approval

This analysis did not involve human subjects, so ethical approval was not required.

## 3 Results

### 3.1 Descriptive characteristics

On average, counties in the study sample had a poverty rate of 12.8% and racial segregation index of 37.9%, and were 73.5% White, 78.2% urban, and 47.2% Democratic (Table 1). Across the 309 counties, mean overall policy comprehensiveness scores ranged from 4.9 to 16.0 (mean 10.8, standard deviation 2.5) (Table 2).

**Table 1:** Characteristics of the 309 counties in the UCCP database.

Characteristics	Mean	SD	Minimum	Maximum
Poverty rate %	12.76	4.74	2.70	30.10
White race %	73.45	16.63	14.40	98.10
Urban area %	78.22	24.89	0	100.00
Racial segregation index %	37.92	11.91	4.96	68.31
Democratic voter %	47.17	17.21	10.00	90.90
Average temperature (°F)	57.32	8.93	39.50	79.30
Primary care physicians per 10 million	0.77	0.32	0	1.88
Manufacturing employment per 100,000	0.22	0.35	0	3.69

UCCP, U. S. COVID-19 County Database; SD, Standard deviation.

**Table 2:** Summary statistics of mean county policy comprehensiveness scores, by policy domain and overall.

Mean policy comprehensiveness score	Mean	SD	Minimum	Maximum
Containment/Closure	3.87	1.15	1.25	7.91
Economic support	3.06	0.90	0.93	4.67
Public health	3.90	0.74	1.82	5.38
Overall	10.83	2.48	4.90	15.95

*N* = 309 counties in 50 states and Washington, D. C. drawn from the U. S. COVID-19 County Database. SD, Standard deviation.

### 3.2 Association of county characteristics with policy comprehensiveness

Table 3 presents the association of county characteristics with county-level mean policy comprehensive scores, by policy domain and overall. Notably, the units of the county characteristics varied and therefore the corresponding coefficients are not directly comparable. While White race was positively associated with economic support policy comprehensiveness (0.01 per 1% increase in White race, 95% CI 0.00, 0.02), the number of primary care physicians per 100,000 was negatively associated (−0.40 per 1 primary care physician/ 100,000 people, 95% CI -0.75, −0.05). Urbanicity was positively associated with public health policy comprehensiveness (0.01 per 1% increase in urban area, 95% CI 0.00, 0.01). Similarly, Democratic voter percentages had positive associations with all policy domain comprehensiveness scores, while average temperature had negative associations. The number of manufacturing jobs per 100,000 people was positively associated with average containment/closure policy comprehensiveness scores (0.37 per 1 job increase in manufacturing/100,000 people, 95% CI 0.03, 0.71) and overall policy comprehensiveness scores (0.74 per 1 job increase in manufacturing/100,000 people, 95% CI 0.02, 1.46). The R-squared values for regression models ranged from 0.22 to 0.35, suggesting a substantial proportion of the variance in policy comprehensiveness scores was explained by county characteristics.

**Table 3:** Association of county characteristics with mean county policy comprehensiveness scores, by policy domain and overall.

County characteristics	Containment/closure	Economic support	Public health	Overall
	Coefficient^1^ [95% CI] *p-*value			
Poverty rate %	−0.01	−0.02	0.00	−0.02
	[−0.04, 0.02]	[−0.04, 0.00]	[−0.01, 0.02]	[−0.08, 0.04]
	0.62	0.11	0.64	0.46
White race %	0.00	0.01*	0.01	0.02
	[−0.01, 0.01]	[0.00, 0.02]	[−0.00, 0.01]	[−0.00, 0.04]
	0.82	0.02	0.07	0.11
Urban area %	0.01	0.00	0.01**	0.02
	[−0.00, 0.01]	[−0.00, 0.01]	[0.00, 0.01]	[−0.00, 0.03]
	0.10	0.40	0.01	0.05
Racial segregation index %	−0.01	0.00	−0.00	−0.00
	[−0.02, 0.01]	[−0.01, 0.01]	[−0.01, 0.01]	[−0.03, 0.02]
	0.42	0.63	0.85	0.81
Democratic voter %	0.03***	0.02***	0.02***	0.07***
	[0.02, 0.04]	[0.01, 0.03]	[0.01, 0.03]	[0.05, 0.09]
	0.00	0.00	0.00	0.00
Average temperature (°F)	−0.03***	−0.02***	−0.02***	−0.07***
	[−0.05, -0.02]	[−0.03, -0.01]	[−0.02, -0.01]	[−0.10, -0.04]
	0.00	0.00	0.00	0.00
Primary care physicians per 10 million	−0.25	−0.40*	−0.07	−0.73
	[−0.67, 0.17]	[−0.75, -0.05]	[−0.33, 0.19]	[−1.60, 0.15]
	0.24	0.02	0.59	0.10
Manufacturing employment per 100,000	0.37*	0.23	0.14	0.74*
	[0.03, 0.71]	[−0.06, 0.51]	[−0.08, 0.36]	[0.02, 1.46]
	0.03	0.12	0.20	0.05
R-squared	0.315	0.221	0.340	0.351

*N* = 309 counties in 50 states and Washington, D. C. drawn from the U. S. COVID-19 County Database. Values in each cell represent coefficients from multivariable regressions. CI Confidence interval.

*p < 0.05; **p < 0.01; ***p < 0.001.

^1^Coefficients represent the change in comprehensiveness score for every 1 unit increase in the predictor. For “%” predictors, 1% is equivalent to 1 unit. For temperature, 1 unit is 1°F. For primary care physicians, 1 unit represents 1 primary care physician per 10 million people. For manufacturing employment, 1 unit represents 1 part- or full-time job in manufacturing per 100,000 people.

### 3.3 Sensitivity analysis

Results of sensitivity analyses using maximum, as opposed to mean, policy comprehensiveness scores revealed similar associations between Democratic voter percentages, average temperature, and manufacturing employment per 100,000 and overall policy comprehensiveness scores (Table 4). However, manufacturing employment per 100,000 was newly positively associated with maximum economic support policy comprehensiveness (0.30 per 1 job increase in manufacturing/100,000 people, 95% CI 0.05, 0.55), and White race was newly positively associated with maximum overall policy comprehensiveness (0.043 per 1% increase in White race, 95% CI 0.02, 0.06). The number of primary care physicians was newly negatively associated with maximum overall policy comprehensiveness scores (−1.22 per 1 primary care physician increase/ 100,000 people, 95% CI −2.12, −0.31). Urbanicity was again positively associated with maximum public health comprehensiveness scores (0.01 per 1% increase in urban area, 95% CI 0.00, 0.01). In our data, percent urban and population density were highly correlated, therefore, we did not include both variables simultaneously due to collinearity.

**Table 4:** Association of county characteristics with maximum policy comprehensiveness scores, by policy domain and overall.

County characteristics	Containment/closure	Economic support	Public health	Overall
	Coefficient^1^ [95% CI] *p*-value			
Poverty rate %	−0.01	−0.01	0.01	−0.03
	[−0.04, 0.02]	[−0.03, 0.01]	[−0.02, 0.03]	[−0.09, 0.03]
	0.52	0.22	0.91	0.34
White race %	0.01	0.01	0.01	0.04***
	[−0.00, 0.02]	[−0.00, 0.01]	[−0.00, 0.02]	[0.02, 0.06]
	0.08	0.05	0.07	0.00
Urban area %	0.00	0.00	0.01*	0.01
	[−0.00, 0.01]	[−0.00, 0.01]	[0.00, 0.01]	[−0.01, 0.02]
	0.34	0.63	0.02	0.34
Racial segregation index %	0.00	0.01	−0.00	0.00
	[−0.01, 0.01]	[−0.00, 0.01]	[−0.01, 0.01]	[−0.03, 0.03]
	0.52	0.30	0.88	0.99
Democratic voter %	0.02***	0.02***	0.03***	0.10***
	[0.01, 0.03]	[0.01, 0.03]	[0.02, 0.03]	[0.07, 0.12]
	0.00	0.00	0.00	0.00
Average temperature (°F)	0.00	−0.03***	−0.02***	−0.04*
	[−0.01, 0.02]	[−0.04, -0.02]	[−0.04, -0.01]	[−0.07, -0.01]
	0.54	0.00	0.00	0.02
Primary care physicians per 10 million	−0.40	−0.25	−0.13	−1.22**
	[−0.76, 0.02]	[−0.55, 0.05]	[−0.49, 0.23]	[−2.12, -0.31]
	0.06	0.11	0.47	0.01
Manufacturing employment per 100,000	0.23	0.30*	0.20	1.06**
	[−0.09, 0.55]	[0.05, 0.55]	[−0.10, 0.49]	[0.31, 1.80]
	0.17	0.02	0.19	0.01
R-squared	0.164	0.264	0.298	0.329

*N* = 309 counties in 50 states and Washington, D. C. drawn from the U. S. COVID-19 County Database. Values in each cell represent coefficients from multivariable regressions. CI, Confidence interval.

*p < 0.05; **p < 0.01; ***p < 0.001.

^1^Coefficients represent the change in comprehensiveness score for every 1 unit increase in the predictor. For “%” predictors, 1% is equivalent to 1 unit. For temperature, 1 unit is 1°F. For primary care physicians, 1 unit represents 1 primary care physician per 10 million people. For manufacturing employment, 1 unit represents 1 part- or full-time job in manufacturing per 100,000 people.

## 4 Discussion

Our study is among the first to explore factors associated with substantial variation in local policymaking across U. S. counties during the pandemic, using a robust and novel county-level policy database. The results suggest that several structural and political factors were associated with policy comprehensiveness across multiple domains. These results add unique insights into state-level responses demonstrate similar patterns that were evident at the county level by highlighting how hyper-local context may shape decision-making during public health emergencies.

A consistent finding was that there were more comprehensive policies—overall and related to containment/closure and economic policies—in counties with more Democratic voters. This pattern is consistent with prior studies that have found Democrats reported more concerns about the pandemic (28). Governors and local officials may have been more responsive to these concerns, prioritizing public safety and marginalized populations. In another analysis of U. S. governors’ stay-at-home orders, Democratic governors were significantly more likely to implement statewide stay-at-home orders (29). Similarly, school districts in counties with higher Democratic presidential vote shares were significantly less likely to plan for in-person reopening and more likely to adopt all-virtual learning plans (20). Other studies have shown that Republican-controlled states issued lockdowns later, were less likely to mandate mask-wearing, and began to reopen the economy earlier (14). While our analyses are correlational, and cannot establish causality, the findings suggest that county political context was associated with pandemic-era policy comprehensiveness. Prior work suggests that such policy differences may have downstream implications for disparities in individual health behaviors and outcomes (30).

Urbanicity positively associated with public health, and overall policy comprehensiveness, suggesting population density may have shaped the earlier stages in which containment and mitigation measures were enacted. Urban counties may face greater pressure to adopt visible public health measures because of complex service infrastructures, and greater perceived risk of transmission. In sensitivity analyses, substituting population density for percent urban yielded similar associations, but the high correlation between these measures precluded their simultaneous inclusion in the same models. This suggests that urbanicity and density capture related features of local context relevant to policy response. Another notable finding was the negative association between primary care physician supply and comprehensiveness scores. Counties with more PCPs per capita had less comprehensive policies overall and for certain domains. One possible interpretation is the counties with greater clinical capacity may have relied more heavily on the healthcare system to manage COVID-19 rather than implementing broad preventative public health policies. Alternatively, PCP supply may also be acting as a proxy for other county characteristics not fully captured in our models. This interpretation is broadly consistent with another study showing that stringent polices were most effective in counties with fewer physicians (31). Similarly, manufacturing employment was associated with greater policy comprehensiveness, particularly for containment and closure policies. This pattern may reflect the concentration of workers in settings perceived to be at higher risk for transmission, although the present analysis cannot identify the specific nuances. We also found reduced comprehensiveness of economic policies in counties with higher poverty rates, suggesting that communities with fewer resources may have faced greater constraints developing or implementing robust economic protections. This pattern risks exacerbating pre-pandemic health disparities, as counties with greater need may have had fewer tools to mitigate the economic and health consequences of the pandemic. Such gaps in support may also have increased vulnerability to transmission and downstream social harms. Federal initiatives such as the American Rescue Plan (32), provided to state and local governments, implemented a range of economic measures including, small-businesses loans (33), medical debt relief, enacted eviction prevention policies (13, 34), and investments in water and sewer infrastructure in high poverty areas (35). Although relatively few studies have examined their impact, emerging evidence suggests that such supports can reduce COVID-19 infections and deaths (34) demonstrating the potential importance of comprehensive economic policies during public health emergencies.

Our findings suggest that future emergency funding strategies may benefit from strengthening local capacity to enact comprehensive economic and public health policies, particularly in counties facing structural disadvantage. This study also helps bridge state- and local-level policy research. By incorporating state-level policies when county policies were unavailable, our outcome measures effectively reflect a combination of state policy baselines and county tailored responses to local needs. Future multilevel analyses could extend this work by explicitly modeling state-level predictors alongside county characteristics to disentangle these cross-level influences.

We intentionally focused on pre-pandemic county characteristics rather than including time-varying pandemic indicators such as local COVID-19 case rates or vaccination coverage. These factors varied substantially in the latter part of our study period and were themselves likely influenced by policy and local context. Finally, we emphasize that greater policy comprehensiveness does not imply “better” policymaking. In some cases, more comprehensive containment and closure policies may have achieved reduced pandemic control while also contributing to social isolation, employment disruption, or economic hardship if not paired with adequate supports. Our analysis was not designed to evaluate the health or social impacts of specific policy combinations, but rather to identify count-level predictors of comprehensiveness. Future research could integrate policy measures such as those used here to assess how local policy environments shape the broader consequences of public health emergencies.

This study is not without limitations. First, it is cross-sectional and observational; accordingly the estimated associations between county characteristics and policy comprehensiveness should not be interpreted causally. Second, we used county-level data, and did not examine explicitly model state-level variation, which was beyond the scope of the current analysis. Third, our study period was restricted to the first two years of the pandemic. County characteristics that may have been important predictors of comprehensiveness early in the pandemic may have differed from those in later phases as vaccination increased, and public fatigue with restrictions grew. Fourth, we did not include all U. S. counties in the dataset, although the current analytic sample included 309 counties across multiple states and represented over half of the U. S. population. Counties were selected using a complex systematic process, and because survey weights were not available, our analyses are not nationally representative of all U. S. counties; results should be interpreted as generalizable only to the sampled counties. Fifth, we did not examine the effects of these policies, although prior studies have examined the impact of pandemic-related policies on outcomes such as population mobility, (36) school closures (20), and pollution (37). Future research should examine how variation in policymaking influenced physical and mental health as well as the racial and social inequities in health during and after the pandemic. Finally, the characteristics examined here represent only a subset of the full range of possible county-level predictors of policy comprehensiveness. Although we selected variables with strong theoretical relevance other political, institutional, and social factors likely also contributed to country policy response.

## 5 Conclusion

In conclusion, results from this cross-sectional study suggest that several county-level characteristics – particularly political composition, urbanicity, climate, manufacturing employment, and healthcare capacity – were associated with variation in local policymaking during the COVID-19 pandemic. While decentralized public health policymaking in the U. S. reflects constitutional principles and allows for local adaptation, it may also hinder a more unified response to public health crises. By leveraging a unique county-level policy database, we provide an initial national-scale snapshot of how structural and political features of local communities were associated with the scope of their pandemic-era responses.

These findings may have implications beyond the COVID-19 pandemic. Identifying county characteristics associated with more or less comprehensive policy responses can inform other public health emergencies including strengthening policy in economically disadvantaged or resource-limited counties. More broadly, this work highlights the importance of local policy capacity as a potential contributor to geographic variation in emergency response.

Future studies should examine policy predictors beyond the pandemic context to inform research on the roots of geographic health disparities. While we focused here on pandemic-era policies, similar approaches can be applied to other domains such as climate health, housing, and chronic disease prevention. Future work integrating detailed policy data with longitudinal health outcomes may help clarify how local policy environments shape population health over time.

## Data Availability

Publicly available datasets were analyzed in this study. This data can be found at: https://data-catalog.cpr3.ucsf.edu/cpr3/s/rdc-dataset/a0U5w00000fTAbBEAW/ds000369.

## References

[1] Carter DP May PJ. Making sense of the US COVID-19 pandemic response: a policy regime perspective. Adm Theory Prax. (2020) 42:265–77. doi: 10.1080/10841806.2020.1758991

[2] Cigler BA. Fighting COVID-19 in the United States with federalism and other constitutional and statutory authority.Oxford University Press on Behalf of CSF Associates: Publius, Inc. (2021).

[3] Gluck AR. Our national federalism. Yale Law J. (2014) 123:1996–2043.

[4] Kettl DF. States divided: the implications of American federalism for COVID-19. Public Adm Rev. (2020) 80:595–602. doi: 10.1111/puar.13243, 32836439 PMC7280573

[5] National Conference of State Legislatures. State Quarantine and Isolation Statutes. Denver, CO/Washington, DC: National Conference of State Legislatures. (2021).

[6] McDonald BD Goodman CB Hatch ME. Tensions in state–local intergovernmental response to emergencies: the case of COVID-19. State Local Gov Rev. (2020) 52:186–94. doi: 10.1177/0160323X20979826

[7] U.S. Department of Health and Human Services. Who has the Authority to Enforce Isolation and Quarantine because of a Communicable Disease? Washington, DC: U.S. Department of Health and Human Services. (2025).

[8] U.S. Census Bureau. SAIPE State and County estimates for 2019. Washington, DC: U.S. Census Bureau. (2019). Available online at: https://www.census.gov/data/datasets/2019/demo/saipe/2019-state-and-county.html (Accessed January 1, 2023).

[9] Hamad R Lyman KA Lin F Modrow MF Ozluk P Azar KMJ . The US COVID-19 county policy database: a novel resource to support pandemic-related research. BMC Public Health. (2022) 22:2148. doi: 10.1186/s12889-022-14132-6, 36217102 PMC9548418

[10] Benfer EA Vlahov D Long MY Walker-Wells E Pottenger JL Jr Gonsalves G . Eviction, health inequity, and the spread of COVID-19: housing policy as a primary pandemic mitigation strategy. J Urban Health. (2021) 98:1–12. doi: 10.1007/s11524-020-00502-1, 33415697 PMC7790520

[11] Leifheit KM Linton SL Raifman J Schwartz GL Benfer EA Zimmerman FJ . Expiring eviction moratoriums and COVID-19 incidence and mortality. Am J Epidemiol. (2021) 190:2503–10. doi: 10.1093/aje/kwab196, 34309643 PMC8634574

[12] Talic S Shah S Wild H Gasevic D Maharaj A Ademi Z . Effectiveness of public health measures in reducing the incidence of covid-19, SARS-CoV-2 transmission, and covid-19 mortality: systematic review and meta-analysis. BMJ. (2021) 375:n2997. doi: 10.1136/bmj-2021-068302, 34789505 PMC9423125

[13] Torres P Warner M. A policy window for equity? The American rescue plan and local government response. J Urban Aff. (2024). doi: 10.1080/07352166.2024.2365788

[14] Druckman JN Klar S Krupnikov Y Levendusky M Ryan JB. Affective polarization, local contexts and public opinion in America. Nat Hum Behav. (2021) 5:28–38. doi: 10.1038/s41562-020-01012-5, 33230283

[15] Grumbach JM. From backwaters to major policymakers: policy polarization in the states, 1970-2014. Perspect Polit. (2018) 16:416–35. doi: 10.1017/s153759271700425x

[16] Arons A Pomeranz J Hamad R. Identifying novel predictors of state legislative action to address obesity. J Public Health Manag Pract. (2021) 27:E9–E18. doi: 10.1097/phh.0000000000001039, 31415263 PMC7012724

[17] Pomeranz JL Siddiqi A Bolanos GJ Shor JA Hamad R. Consolidated state political party control and the enactment of obesity-related policies in the United States. Prev Med. (2017) 105:397–403. doi: 10.1016/j.ypmed.2017.08.028, 28865810 PMC5653399

[18] Montez J Beckfield J Cooney J Grumbach J Hayward M Koytak H . US state policies, politics, and life expectancy. Milbank Q. (2020) 98:668–99. doi: 10.1111/1468-0009.12469, 32748998 PMC7482386

[19] Rodriguez JM Geronimus AT Bound J Wen R Kinane CM. Partisan control of U.S. state governments: politics as a social determinant of infant health. Am J Prev Med. (2022) 62:1–8. doi: 10.1016/j.amepre.2021.06.007, 34446314 PMC10929005

[20] Grossmann M Reckhow S Strunk KO Turner M. All states close but red districts reopen: the politics of in-person schooling during the COVID-19 pandemic. Educ Res. (2021) 50:637–48. doi: 10.3102/0013189x211048840

[21] Millett GA Jones AT Benkeser D Baral S Mercer L Beyrer C . Assessing differential impacts of COVID-19 on black communities. Ann Epidemiol. (2020) 47:37–44. doi: 10.1016/j.annepidem.2020.05.003, 32419766 PMC7224670

[22] Braveman PA Cubbin C Egerter S Williams DR Pamuk E. Socioeconomic disparities in health in the United States: what the patterns tell us. Washington, DC: American Public Health Association. (2010) 100:S186–96. doi: 10.2105/ajph.2009.166082, 20147693 PMC2837459

[23] Chiang Y Pacca L Vable A Carton T Pletcher M Hamad R. Trajectories and patterns of US counties' policy responses to the COVID-19 pandemic: a sequence analysis approach. SSM Popul Health. (2025) 29:101734. doi: 10.1016/j.ssmph.2024.101734, 39791112 PMC11714380

[24] Hale T Petherick A Anania J, Variation in Government Responses to COVID-19, version 15. Oxford, UK: Blavatnik School of Government, University of Oxford. (2020). Available online at: https://www.bsg.ox.ac.uk/research/publications/variation-government-responses-covid-19 (Accessed January 1, 2023).

[25] Harris R Taylor R Thielke J Payne N Gonzalez J. Conde, research electronic data capture (REDCap) – a metadata-driven methodology and workflow process for providing translational research informatics support. J Biomed Inform. (2009) 42:377–81. doi: 10.1016/j.jbi.2008.08.010 18929686 PMC2700030

[26] Massey DS Denton NA. The dimensions of residential segregation. Soc Forces. (1988) 67:281–315. doi: 10.2307/2579183

[27] U.S. Census Bureau Using 1-year or 5-year American Community Survey data. Washington, DC: U.S. Census Bureau. Available online at: https://www.census.gov/programs-surveys/acs/guidance/estimates.html

[28] Allcott H Boxell L Conway J Gentzkow M Thaler M Yang D. Polarization and public health: partisan differences in social distancing during the coronavirus pandemic. J Public Econ. (2020) 191:104254. doi: 10.1016/j.jpubeco.2020.104254, 32836504 PMC7409721

[29] Baccini L Brodeur A Weymouth S. The COVID-19 pandemic and the 2020 US presidential election. J Popul Econ. (2021) 34:739–67. doi: 10.1007/s00148-020-00820-3, 33469244 PMC7809554

[30] Darling-Hammond S Michaels EK Allen AM Chae DH Thomas MD Nguyen TT . After "the China virus" went viral: racially charged coronavirus coverage and trends in Bias against Asian Americans. Health Educ Behav. (2020) 47:870–9. doi: 10.1177/1090198120957949, 32911985 PMC7488172

[31] Sun Y Bisesti E. Political economy of the COVID-19 pandemic: how state policies shape county-level disparities in COVID-19 deaths. Socius. (2023) 9. doi: 10.1177/23780231221149902, 36777497 PMC9902801

[32] U.S. Department of the Treasury. Assistance for State, Local, and Tribal Governments. Washington, DC: U.S. Department of the Treasury.

[33] Wilson D Johnson B Stokan E Overton M. Institutional collective action during COVID-19: lessons in local economic development. Public Adm Rev. (2020) 80:862–5. doi: 10.1111/puar.13234, 32836454 PMC7300681

[34] Jowers K Timmins C Bhavsar N Hu Q Marshall J. Housing Precarity and the COVID-19 Pandemic: Impacts of Utility Disconnection and Eviction Moratoria on Infections and Deaths Across US Counties. In: Working Paper Series. Cambridge, MA: National Bureau of Economic Research (2021).

[35] Kang YJ Turlapati L Schwab J Huang J. Water and Sewer Infrastructure: Correcting Underinvestment with Smart Spending. Ithaca, NY: Department of City and Regional Planning, Cornell (2022).

[36] Feyman Y Bor J Raifman J Griffith KN. Effectiveness of COVID-19 shelter-in-place orders varied by state. PLoS One. (2020) 15:e0245008. doi: 10.1371/journal.pone.0245008, 33382849 PMC7775080

[37] Li Y Li MM Rice M Yang CW. Impact of COVID-19 containment and closure policies on tropospheric nitrogen dioxide: a global perspective. Environ Int. (2022) 158. doi: 10.1016/j.envint.2021.106887, 34563750 PMC8452510

[38] Bureau C. (2010) Census urban and rural classification and urban area criteria. Available online at: https://www.census.gov/programs-surveys/geography/guidance/geo-areas/urban-rural/2010-urban-rural.html (Accessed January 1, 2023).

