# Supplementary material for: A cross-sectional analysis of county-level determinants of pandemic-era policy implementation
Source: Front Public Health. 2026 Jun 19;14:1797664. doi: 10.3389/fpubh.2026.1797664 (PMC13330766; doi:10.3389/fpubh.2026.1797664)
Supplement: Supplementary file 1 [file Data_Sheet_1.pdf]

SUPPLEMENTAL MATERIALS

Supplemental Methods

To assess the accuracy of the policy data collection process, a second round of data collection included a subset of 10 counties randomly selected from the original round of 100 counties. Six data collectors were randomly assigned to complete the second entry of these 13 records using the same process as for the original data collection. Data collectors were excluded from collecting the same record twice. Primary and secondary data collectors assessed 13 records across 75 weeks. As each collection (original and secondary rounds) included 26 indicators for county surveys, the overlap sample included over 25,000 data points for comparison and analysis (13 records x 26 indicators per county record x 75 weeks).

To estimate interrater reliability for the first versus second round of data collection, we first calculated the percentage of agreement between the two data entries, excluding any data for which there was missingness in either entry. Second, we calculated the kappa statistic for interrater reliability, which estimated the extent to which the data collected by different raters (in our case, two different data entries) agreed on interpreting the policy indicators measured. **Supplemental Table D** provides the kappa statistics by county across each indicator. Kappa statistics represent the average agreement beyond chance across the set of rater pairs.

Supplemental Table A. List of counties included in US COVID-19 County Policy Database

State	County	State	County	State	County	State	County
Alabama	Covington	Georgia	Bartow	Nevada	Clark	South Carolina	Beaufort
Alabama	Cullman	Georgia	Chatham	New Hampshire	Hillsborough	South Carolina	Berkeley
Alabama	Etowah	Georgia	Cobb	New Hampshire	Merrimack	South Carolina	Dorchester
Alabama	Jefferson	Georgia	Fulton	New Jersey	Atlantic	South Carolina	Greenville
Alabama	Lee	Georgia	Gwinnett	New Jersey	Bergen	South Carolina	Lee
Alabama	Limestone	Georgia	Lowndes	New Jersey	Burlington	South Carolina	Oconee
Alabama	Mobile	Georgia	Macon	New Jersey	Camden	South Carolina	Richland
Alabama	Montgomery	Georgia	Richmond	New Jersey	Cumberland	South Carolina	Spartanburg
Alaska	Aleutians West	Georgia	Thomas	New Jersey	Essex	South Carolina	York
Arizona	Mohave	Georgia	DeKalb	New Jersey	Gloucester	South Dakota	Yankton
Arizona	Navajo	Hawaii	Honolulu	New Jersey	Hudson	Tennessee	Hamilton
Arizona	Maricopa	Idaho	Bingham	New Jersey	Mercer	Tennessee	Hawkins
Arizona	Pima	Idaho	Canyon	New Jersey	Ocean	Tennessee	Lawrence
Arkansas	Ouachita	Idaho	Ada**	New Jersey	Passaic	Tennessee	Shelby
Arkansas	Washington	Illinois	Grundy	New Jersey	Union	Tennessee	Tipton
California	Inyo	Illinois	Jackson	New Mexico	Bernalillo	Tennessee	Wilson
California	Kern	Illinois	Morgan	New Mexico	McKinley	Tennessee	Davidson
California	Madera	Illinois	Will	New Mexico	Santa Fe	Texas	Anderson
California	San Bernardino	Illinois	Winnebago	New York	Erie	Texas	Brazoria
California	Shasta	Illinois	Cook	New York	Monroe	Texas	Brewster
California	Tuolumne	Illinois	DuPage	New York	Onondaga	Texas	Cameron
California	Alameda	Illinois	Kane	New York	Wayne	Texas	El Paso
California	Fresno	Illinois	Lake	New York	Bronx	Texas	Harrison
California	Los Angeles	Indiana	Allen	New York	Kings	Texas	Hidalgo
California	Orange	Indiana	Huntington	New York	Nassau	Texas	Johnson
California	Riverside	Indiana	Jennings	New York	New York	Texas	Lavaca

California	Sacramento	Indiana	Lake	New York	Queens	Texas	McLennan
California	San Diego	Indiana	Marion	New York	Suffolk	Texas	Montgomery
California	San Francisco	Indiana	St. Joseph	New York	Westchester	Texas	Nueces
California	San Joaquin	Iowa	Johnson	North Carolina	Catawba	Texas	Palo Pinto
California	Santa Clara	Iowa	Lee	North Carolina	Craven	Texas	Randall
California	Solano	Kansas	Wyandotte	North Carolina	Guilford	Texas	Smith
California	Sonoma	Kansas	Johnson	North Carolina	Iredell	Texas	Bell
California	Stanislaus	Kentucky	Jefferson	North Carolina	Johnston	Texas	Bexar
California	Placer	Kentucky	Warren	North Carolina	Mecklenburg	Texas	Collin
California	Contra Costa	Louisiana	Caddo	North Carolina	Onslow	Texas	Dallas
California	Marin	Louisiana	Calcasieu	North Carolina	Orange	Texas	Harris
California	Napa	Louisiana	Lafayette	North Carolina	Pender	Texas	Tarrant
California	San Mateo	Louisiana	Livingston	North Carolina	Vance	Texas	Denton
California	Santa Clara	Louisiana	St. James	North Carolina	Wake	Texas	Travis
California	El Dorado	Louisiana	Tangipahoa	North Carolina	Chatham	Texas	Brazos
California	Humboldt	Louisiana	East Baton Rouge	North Dakota	Golden Valley	Texas	Williamson
California	Monterey	Louisiana	Jefferson	Ohio	Geauga	Utah	Grand
California	Nevada	Louisiana	Orleans	Ohio	Hamilton	Utah	Salt Lake
California	San Luis Obispo	Louisiana	St. Tammany	Ohio	Montgomery	Utah	Utah
California	Santa Cruz	Maine	Washington	Ohio	Portage	Utah	Davis
California	Ventura	Maryland	Baltimore City	Ohio	Scioto	Vermont	Chittenden
Colorado	Douglas	Maryland	Prince George's	Ohio	Cuyahoga	Virginia	Chesapeake City
Colorado	El Paso	Maryland	Montgomery	Ohio	Franklin	Virginia	Dickenson
Colorado	Huerfano	Maryland	Baltimore	Oklahoma	Bryan	Virginia	Fredericksburg City
Colorado	Mesa	Massachusetts	Hampden	Oklahoma	Oklahoma	Virginia	Orange
Colorado	Adams	Massachusetts	Essex	Oklahoma	Pushmataha	Virginia	Patrick
Colorado	Arapahoe	Massachusetts	Middlesex	Oklahoma	Tulsa	Virginia	Virginia Beach City
Colorado	Boulder	Massachusetts	Norfolk	Oregon	Clatsop	Virginia	Arlington
Colorado	Denver	Massachusetts	Suffolk	Oregon	Coos	Virginia	Fairfax
Connecticut	Hartford	Massachusetts	Worcester	Oregon	Jackson	Washington	Clark

Connecticut	Middlesex	Michigan	Berrien	Oregon	Linn	Washington	Kitsap
Connecticut	New Haven	Michigan	Genesee	Oregon	Marion	Washington	Lewis
Connecticut	Fairfield	Michigan	Macomb	Oregon	Wallowa	Washington	Pierce
Delaware	New Castle	Michigan	Roscommon	Oregon	Washington	Washington	Skagit
DC	DC	Michigan	Oakland	Oregon	Clackamas	Washington	Spokane
Florida	Broward	Michigan	Wayne	Oregon	Multnomah	Washington	Yakima
Florida	Duval	Michigan	Washtenaw	Pennsylvania	Berks	Washington	King
Florida	Hernando	Minnesota	Ramsey	Pennsylvania	Erie	Washington	Snohomish
Florida	Hillsborough	Minnesota	Hennepin	Pennsylvania	Jefferson	West Virginia	Cabell
Florida	Indian River	Mississippi	Grenada	Pennsylvania	Lackawanna	Wisconsin	Grant
Florida	Lake	Mississippi	Lowndes	Pennsylvania	Lebanon	Wisconsin	Oconto
Florida	Lee	Mississippi	Simpson	Pennsylvania	Monroe	Wisconsin	St. Croix
Florida	Manatee	Mississippi	Union	Pennsylvania	Schuylkill	Wisconsin	Taylor
Florida	Miami-Dade	Missouri	Boone	Pennsylvania	Wayne	Wisconsin	Milwaukee
Florida	Orange	Missouri	Jackson	Pennsylvania	Westmoreland	Wisconsin	Brown
Florida	Osceola	Missouri	Pettis	Pennsylvania	Allegheny	Wisconsin	Dane
Florida	Palm Beach	Missouri	Phelps	Pennsylvania	Philadelphia	Wisconsin	Kenosha
Florida	Pasco	Missouri	Shannon	Pennsylvania	Montgomery	Wisconsin	Ozaukee
Florida	Pinellas	Missouri	St. Louis City	Rhode Island	Providence	Wisconsin	Racine
Florida	Polk	Missouri	St. Louis	Rhode Island	Washington	Wisconsin	Sheboygan
Florida	Santa Rosa	Montana	Musselshell			Wyoming	Natrona
Florida	Volusia	Nebraska	Douglas				

Note: Data were drawn from the US COVID-19 County Policy Database across 101 weeks from January 2020 to December 2021 (N = 309 counties across 50 states and Washington DC).

Supplemental Table B. Missing County Characteristics

Variable	Missing	Total	Percent Missing
FIPS code	0	309	0
County Name	0	309	0
State Abbreviation	0	309	0
Poverty Rate	0	309	0
SVI Socioeconomic	0	309	0
SVI Household Composition & Disability	0	309	0
SVI Minority Status & Language	0	309	0
SVI Housing Type & Transportation	0	309	0
SVI Overall Vulnerability	0	309	0
HPSA Code	0	309	0
Primary Care Physicians Rate	2	309	0.65
GDP	0	309	0
Farming	0	309	0
Manufacturing	0	309	0
Accommodation and Food Services	0	309	0
Unemployment rate	0	309	0
Average Temperature	0	309	0
Precipitation	0	309	0
Population	0	309	0

Population Density	0	309	0
White	0	309	0
Black or African American	0	309	0
Asian Americans and Pacific Islanders	0	309	0
Other race	0	309	0
Hispanic	0	309	0
Education Attainment	0	309	0
Household Overcrowding	0	309	0
Income	0	309	0
Urban Areas %	0	309	0
Racial dissimilarity Index	1	309	0.32
Proportion of Democratic vote	0	309	0

Supplemental Table C. Pairwise correlations between county characteristics

Characteristics	Poverty Rate	White Race	Urban Area %	Racial Segregation	Democratic Voter %	Average Temperature	Primary Care Physician Rate	Manufacturing Employment
Poverty Rate %	1.00							
White Race %	-0.27	1.00						
Urban Area %	-0.26	-0.36	1.00					
Racial Segregation Index %	0.20	-0.44	0.50	1.00				
Democratic Voter %	0.02	-0.65	0.62	0.50	1.00			
Average Temperature (°F)	0.25	-0.30	0.14	0.06	-0.01	1.00		
Primary Care Physicians per 10 million	-0.21	-0.11	0.44	0.21	0.48	-0.17	1.00	
Manufacturing Employment per 100,000	-0.14	-0.21	0.37	0.31	0.30	0.01	0.16	1.00

Note: *N* = 309 counties in 50 states (including Washington, D.C.), drawn from the US COVID-19 County Policy Database from January 2020 to December 2021. Values in each cell represent Pearson correlation coefficients between each set of county characteristics.

Examining correlations between each pair of county characteristics (Supplemental Table B), we found several positive moderate (0.4-0.59) correlations between Democratic voter percentage and racial segregation, Democratic voter percentage and primary care physicians, urban area percentage and racial segregation, and urban area percentage and primary care physicians. We also found a strong (>0.6) correlation between Democratic voter percentage and urban area percentage. There were moderate to strong negative correlations between White race and both racial segregation and Democratic voter percentage.

Supplemental Table D. Kappa statistics by county across each indicator.

Table D 1 Kappa statistics by county across each indicator

Variable	Kappa	CI	N
School closing (0-3)	0.47	(0.451 - 0.473)	974
Workplace closing (0-3)	0.738	(0.721 - 0.744)	969
Cancel public events (0-4)	0.666	(0.658 - 0.702)	970
Restrictions on private gathering (0-4)	0.625	(0.608 - 0.631)	974
Close public transport (0-2)	0.353	(0.311 - 0.395)	974
Stay at home requirements (0-3)	0.642	(0.637 - 0.677)	975
Gym closing (0-3)	0.869	(0.867 - 0.872)	973
Restaurant closing (0-5)	0.826	(0.814 - 0.840)	975
Bar closing (0-5)	0.748	(0.744 - 0.759)	975
Movie theater closing (0-3)	0.768	(. - .)	975
Day care closing (0-3)	0.649	(0.640 - 0.666)	975
Restrictions on religious gatherings (0-3)	0.782	(0.765 - 0.802)	975
Curfew requirement (0-1)	0.769	(0.708 - 0.830)	975
Income support (0-3)	0.685	(0.622 - 0.700)	975
Housing support (0-1)	0.75	(0.709 - 0.790)	975
Utility support (0-1)	0.631	(0.582 - 0.681)	975
Paid sick leave (0-1)	1	(1.000 - 1.000)	975
Nutrition support (0-1)	0.681	(0.608 - 0.753)	825
Public information campaigns (0-3)	0.427	(0.411 - 0.467)	975
Testing policy (0-2)	0.587	(. - .)	975
Contact tracing (0-3)	0.57	(0.553 - 0.591)	975
Facial coverings (indoors) (0-3)	0.841	(0.819 - 0.873)	975

Facial coverings (outdoors) (0-3)	0.566	(0.530 - 0.596)	975
Vaccination availability by group (0-1)	0.847	(0.812 - 0.881)	975
Vaccination availability by organization/location (0-1)	0.96	(0.941 - 0.978)	975
Space/event access restrictions (0-3)	-0.005	(-0.012 - 0.001)	928

Values less than 0 indicate less agreement than by chance, and are likely due to nearly all observations falling in the same category.

Table D 2 Percent interrater agreement by county across each indicator.

Variable	% agreement
School closing (0-3)	62.36
Workplace closing (0-3)	81.53
Cancel public events (0-4)	75.57
Restrictions on private gathering (0-4)	75.05
Close public transport (0-2)	62.42
Stay at home requirements (0-3)	78.46
Gym closing (0-3)	90.75
Restaurant closing (0-5)	87.18
Bar closing (0-5)	81.23
Movie theater closing (0-3)	83.59
Day care closing (0-3)	79.18
Restrictions on religious gatherings (0-3)	85.44
Curfew requirement (0-1)	90.87
Income support (0-3)	86.15
Housing support (0-1)	87.59
Utility support (0-1)	82.26
Paid sick leave (0-1)	100
Nutrition support (0-1)	92.48
Public information campaigns (0-3)	74.87
Testing policy (0-2)	76.72
Contact tracing (0-3)	71.59
Facial coverings (indoors) (0-3)	85.85
Facial coverings (outdoors) (0-3)	70.26
Vaccination availability by group (0-1)	92.82
Vaccination availability by organization/location (0-1)	98.15
Space/event access restrictions (0-3)	97.95

Supplemental Table E. Sensitivity analysis of the association of county characteristics with mean county policy comprehensiveness scores, by policy domain and overall

County Characteristics	Containment/Closure	Economic Support	Public Health	Overall
	Coefficient[1] [95% CI] p-value			
Poverty Rate %	-0.01	-0.02	0.00	-0.02
	[-0.04,0.02]	[-0.04,0.00]	[-0.01,0.02]	[-0.08,0.04]
	0.62	0.10	0.61	0.46
White Race %	0.00	0.01*	0.01	0.02
	[-0.01,0.01]	[0.00,0.02]	[-0.00,0.01]	[-0.00,0.04]
	0.81	0.02	0.09	0.11
Urban Area %	0.01	0.00	0.01**	0.02
	[-0.00,0.01]	[-0.00,0.01]	[0.00,0.01]	[-0.00,0.03]
	0.10	0.40	0.01	0.05
Population Density	0.00	0.00	-0.00	0.00
	[-0.00,0.00]	[-0.00,0.00]	[-0.00,0.00]	[-0.00,0.00]
	0.84	0.31	0.11	0.98
Racial Segregation Index %	-0.01	0.00	-0.00	-0.00
	[-0.02,0.01]	[-0.01,0.01]	[-0.01,0.01]	[-0.03,0.02]
	0.42	0.69	0.95	0.81
Democratic Voter %	0.03***	0.02***	0.02***	0.07***
	[0.02,0.04]	[0.01,0.03]	[0.01,0.03]	[0.05,0.10]
	0.00	0.00	0.00	0.00
Average Temperature (°F)	-0.03***	-0.02***	-0.02***	-0.07***
	[-0.05,-0.02]	[-0.03,-0.01]	[-0.03,-0.01]	[-0.10,-0.04]
	0.00	0.00	0.00	0.00
Primary Care Physicians per 10 million	-0.25	-0.40*	-0.07	-0.73
	[-0.67,0.17]	[-0.75,-0.05]	[-0.34,0.19]	[-1.60,0.15]
	0.24	0.03	0.58	0.10
Manufacturing Employment per 100,000	0.37*	0.24	0.13	0.74*
	[0.03,0.72]	[-0.05,0.52]	[-0.09,0.34]	[0.01,1.46]
	0.03	0.11	0.24	0.05
Constant	4.23**	2.78**	2.93**	9.94**
	[2.65,5.81]	[1.45,4.10]	[1.94,3.92]	[6.61,13.3]
	0.00	0.00	0.00	0.00
R-squared	0.315	0.224	0.346	0.351

Note: *N* = 309 counties in 50 states and Washington, D.C. drawn from the U.S. COVID-19 County Database. Values in each cell represent coefficients from multivariable regressions. *CI* Confidence interval.

* p < 0.05; ** p < 0.01; *** p < 0.001

[1] Coefficients represent the change in comprehensiveness score for every 1 unit increase in the predictor. For "%" predictors, 1% is equivalent to 1 unit. For temperature, 1 unit is 1°F. For primary care physicians, 1 unit represents 1 primary care physician per 10 million people. For manufacturing employment, 1 unit represents 1 part- or full-time job in manufacturing per 100,000 people.
